# Simple Method of Controlling Epistaxis

**Published:** 1891-08

**Authors:** 


### Simple Method for Controlling Epistaxis.

Dr. W. W. Parker, of Richmond, Va., says in the New York *Medical Record*: The plan of arresting hæmorrhage from the nose, which I here describe, I have used for thirty years without one failure. When I first began to practice I used Bellocq's instrument but found it painful, and in small children exceedingly troublesome of application. The little device which I use is made of fifteen of the long threads of patent lint, size three and one-half or four inches long, which I double on themselves and tie in the middle, and let one end of the string be six or eight inches long so as to pull the plug out when necessary. When doubled on itself it looks like a "comet" in miniature with a nucleus and thirty tails, or twice the number of threads used. A probe is pressed up against the centre and is passed back upon the floor of the nasal cavity and pushed on till you reach the posterior nares. This will be known both by the resistance and the length of the probe, or the depth which you have reached. Then slowly withdraw the probe and plug the anterior nares and you have arrested the bleeding. These twenty or thirty ends floating in the blood at once coagulate it. The passage of the soft lint gives no pain whatever. If lint is not at hand I use the largest size spool cotton. The plug is removed in from twenty-four to forty-eight hours. It gives no pain and the patient is willing for it to remain. The other methods are all painful in execution, and the discomfort while the plug remains is very considerable.

